# Visions for the North Sea: The Societal Dilemma Behind Specifying Good Environmental Status

**DOI:** 10.1007/s13280-014-0536-5

**Published:** 2014-05-24

**Authors:** Alison J. Gilbert, Abigail McQuatters-Gollop, Olivia Langmead, Laurence Mee, Jan Vermaat

**Affiliations:** 1 Institute for Environmental Studies, VU University Amsterdam, De Boelelaan 1087, 1081 HV Amsterdam, The Netherlands; 2 Sir Alister Hardy Foundation for Ocean Science, The Laboratory, Citadel Hill, Plymouth, PL1 2PB UK; 3 Marine Institute, Plymouth University, Plymouth, PL4 8AA UK; 4 Scottish Association for Marine Science, Scottish Marine Institute, Oban, Argyll, PA37 1QA Scotland, UK; 5 Faculty of Earth and Life Sciences, VU University Amsterdam, De Boelelaan 1087, 1081 HV Amsterdam, The Netherlands

**Keywords:** North Sea, Good Environmental Status, Trawling, Benthic communities, Benthic functioning, Ecosystem services

## Abstract

We augment discussions about the Good Environmental Status of the North Sea by developing two extreme visions and assessing their societal benefits. One vision (‘Then’) assumes restoration of benthic functioning; we contend that trawling had already degraded the southern North Sea a century ago. Available information is used to speculate about benthic functioning in a relatively undisturbed southern North Sea. The second vision (‘Now’) draws on recent benthic functioning. The supply of five ecosystem services, supported by benthic functioning, is discussed. ‘Then’ offers confidence in the sustainable supply of diverse services but restoration of past function is uncertain and likely to be paired with costs, notably trawling restraints. ‘Now’ delivers known and valued services but sustained delivery is threatened by, for example, climate change. We do not advocate either vision. Our purpose is to stimulate debate about what society wants, and might receive, from the future southern North Sea.

**Electronic supplementary material:**

The online version of this article (doi:10.1007/s13280-014-0536-5) contains supplementary material, which is available to authorized users.

## Introduction

North Sea nations are faced with a dilemma as they implement the Marine Strategy Framework Directive (MSFD 2008/56/EC). The North Sea ecosystem is degraded and must be returned to a “Good Environmental Status,” or GES. It suffers from a variety of problems including coastal eutrophication, unsustainable fisheries, and damage to benthic habitats (OSPAR 2010). This paper augments discussions about specifying GES, by developing two extreme visions of the southern North Sea and assessing the potential benefits that they might accord society. We contend that such an approach is needed for three reasons. First, the North Sea, and notably the southern North Sea, was already degraded more than a century ago as a result of the industrialization of demersal trawling in the late-nineteenth century. Degradation pre-dates available data and knowledge. Human activities and associated changes over the last half century only exacerbated this degradation. Second, GES is ultimately a societal choice. An informed choice needs understanding of the benefits that GES would provide (Mee et al. 2008). Third, a vision of what the future might be, supports the development of policy targets.

Demersal trawling disturbs benthic ecosystems. The two visions are contrasted in terms of benthic ecosystem function. One vision corresponds to current and recent functioning. Akin to a business-as-usual or Go with the Flow (Bateman et al. 2014) scenario, we term this the “Now” vision to emphasize that it maintains current functioning—the status quo. The alternative is based on functioning that might have characterized the North Sea prior to degradation and assumes that such functioning can be restored. We call this the “Then” vision to emphasize that it involves restoration of a plausible, past level of functioning. It is not our intention to advocate either vision, but to stimulate a discussion to support decisions about the future of the North Sea environment. We deliberately polarize the discussion by developing and contrasting two extreme visions, and acknowledge that there are any number of intermediates and alternatives.

The two visions are presented in “Visions of the Southern North Sea” section. “Substantiation of the Then Vision” section tracks the industrialization of trawling and our knowledge, both anecdotal and scientific, of the disturbance it has wrought on benthic communities. “Loss of Ecosystem Functioning with Trawling Disturbance” section reconstructs past benthic functioning and “From Benthic Functioning to Ecosystem Services” section identifies associated societal benefits in terms of ecosystem services. “The Societal Choice” section presents the elements inherent in a choice between the two visions.

## Visions of the Southern North Sea

This section presents two visions of the shallow, southern North Sea. We focus on the southern North Sea because it is shallow, has higher concentrations of human populations in its catchment, and a long history of human use. The Now vision is based on its recent, disturbed state and the functioning of its depauperate benthic communities. The Then vision is based on our limited knowledge of how the southern North Sea might have functioned in the mid- to late-nineteenth century prior to the industrialization of trawling (elaborated in “Substantiation of the then Vision” section). We contend that expansion of industrial trawling at the end of the nineteenth century, with widespread disturbance of benthic communities, caused sea-wide degradation. Limits to our knowledge regarding past states means that the Then vision is somewhat speculative.

The two visions are characterized in terms of ecosystem function, a term that is not always clearly defined. We follow Duffy and Stachowicz (2006): ecosystem functions are aggregate ecosystem processes. Drawing from Cochrane et al. (2010), seven benthic functions characterize the two visions (see Table 1): sedimentation, filtration, primary production, secondary production, trophic complexity, nutrient exchange, and recruitment to commercial stocks (functions are further specified in Electronic Supplementary Material Table S1).

**Table 1:** Characterization of two visions of the North Sea on the basis of benthic function

Criterion	Now vision	Then vision
Sedimentation	Reduced function as indicated by relatively turbid water column and mobile sediments, in part due to high rates of demersal trawling^a^	Sediment deposition and stabilization facilitated by benthic communities resulting in a relatively transparent water column^f^
Filtration	Reduced filtration capacity due to loss of epibenthic suspension feeders^b^ (organisms that feeds on particulate organic matter suspended in the water column)^c^	High filtration capacity; relatively high proportion of filter and suspension feeders in benthic communities; abundant coastal beds of *Ostrea edulis* plus a large bed on the Oyster Grounds^b,f,g^
Primary production (PP)	Limited benthic primary production due to turbidity and mobile sediments; pelagic zones highly productive but limited by light availability^d^	Potential for high benthic primary production due to good water transparency; pelagic production probably nutrient-limited^h^
Secondary production	Predominance of deposit feeders, and predators and scavengers feeding on moribund organisms and trawling discards; long-lived species relatively rare^e^	Predominance of epibenthic species, especially suspension feeders that extract particulate organic carbon from water column; long-lived species relatively abundant
Trophic complexity	Simplified benthic food webs; higher proportions of small-bodied, fast-growing, and/or opportunistic species^b^	Complex benthic food webs with a greater abundance of epibenthic; higher proportions of large-bodied and/or long-lived species^i^
Nutrient exchange	Rapid return of nutrients to water column following mineralization by benthic microbes, facilitated by sediment resuspension^e^	Benthic communities intercept nutrients, modulating their return to the water column and keeping pelagic primary productivity nutrient-limited^i^
Recruitment	Species favouring open habitats and/or perturbation regimes recruit best; low diversity of benthic fish species; predominance of fast-growing species	Complex benthic habitats support recruitment by providing nursery habitats; high diversity of benthic fish species includes slow-growing species and long-lived species^f^

^a^Rijnsdorp et al. (1998); ^b^ Rumohr and Kujawski (2000), Tillin et al. (2006), and Callaway et al. (2007); ^c^ http://www.marlin.ac.uk/biotic/imgs/BioticGlossaries.pdf; ^d^ McQuatters-Gollop et al. (2007); ^e^ Artioli et al. (2008) and Vermaat et al. (2008); ^f^ Holt et al. (1998); ^g^ De Vooys et al. (2004); ^h^ Nichols et al. (1990), Stewart and Haynes (1994), and Fahnenstiel et al. (1995); ^i^ Kaiser et al. (2002) and Collie et al. (2000)

**Fig. 1:**
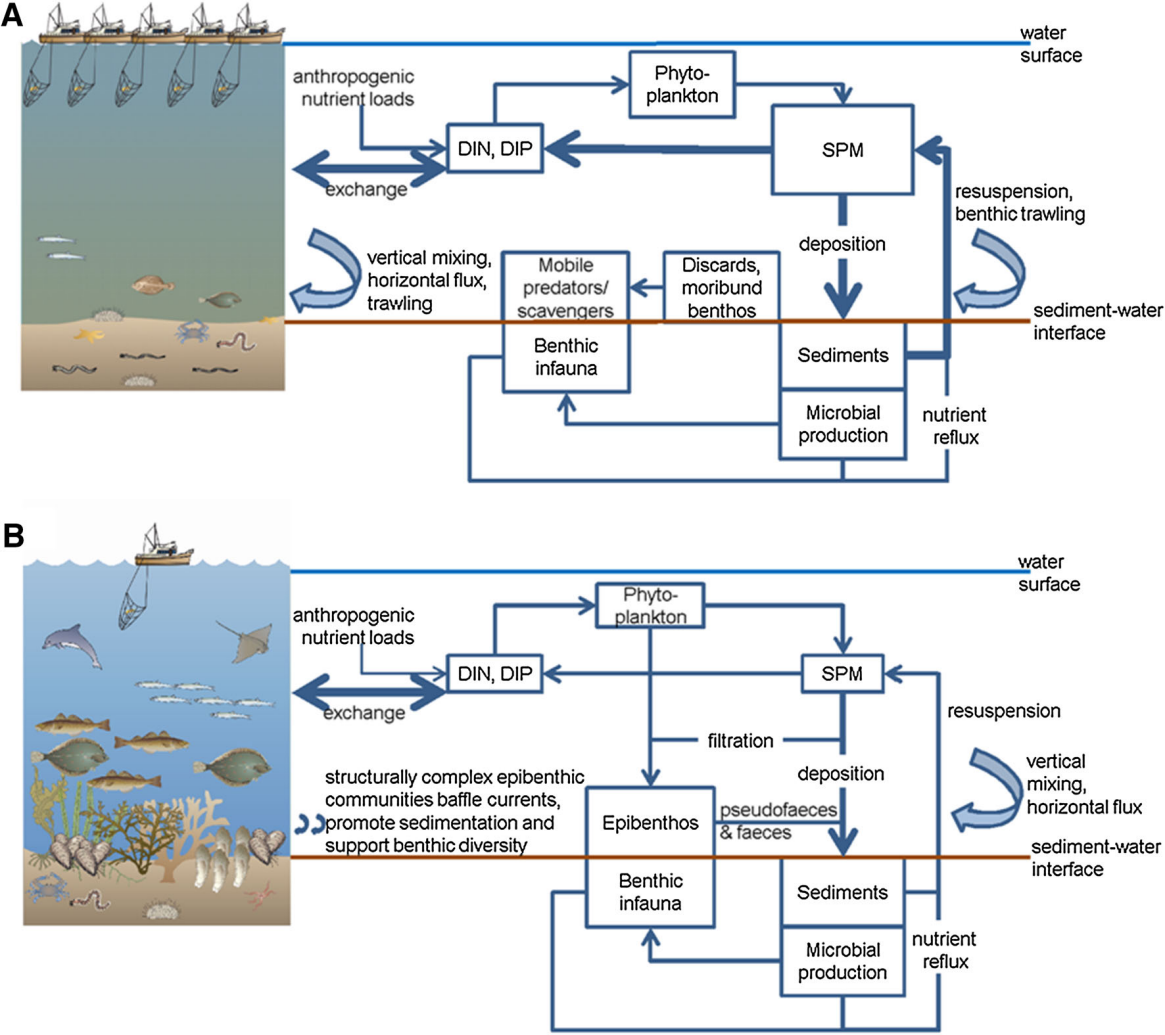
Visions of the coastal/southern North Sea: **A** Now vision characterized by low transparency, high trawling effort, and depauperate benthic communities; **B** Then vision characterized by no trawling, high transparency, and diverse benthic communities

Figure 1 depicts how benthic community functioning affects stocks (rectangles) and flows (arrows) of matter in the two visions. Poor water transparency in the Now vision (Fig. 1A) results from relatively large stocks of suspended particular matter (SPM) and phytoplankton biomass (McQuatters-Gollop et al. 2007). Sediments are readily resuspended with no sedentary epibenthos binding them and baffling water flows, and with extensive and repeated trawling (Rijnsdorp et al. 1998). Benthic communities are dominated by infauna and mobile epibenthos feeding on discards and moribund organisms from trawling (Tillin et al. 2006; Callaway et al. 2007; Rumohr and Kujawski 2000). Nutrient fluxes are dominated by lateral exchange with the open North Sea but are also paired with sediment fluxes associated with deposition and resuspension; nutrient cycling is relatively rapid (Vermaat et al. 2008). In the Then vision (Fig. 1B), diverse epibenthic communities filter the water column removing suspended particulate matter and increasing water transparency. These communities generate habitats with a high structural and trophic complexity. Structural complexity baffles water currents and binds sediments, inhibiting resuspension. Nutrient budgets are still heavily influenced by exchange, but reduced resuspension and interception by benthic communities slows the rate of nutrient reflux.

## Substantiation of the Then Vision

This section summarizes the current state of knowledge regarding trawling’s impacts on benthic communities and presents data and anecdotal information on the industrialization of trawling.

### Current Knowledge of the Adverse Impacts of Fisheries on Benthic Communities

Recent studies clearly show that mobile demersal gears disturb benthic environments by altering seabed morphology and removing, damaging, or killing biota, causing substantial changes in benthic community structure. Trawling removes larger fauna, damages species with fragile shells and tests, and removes and injures long-living species, particularly epifaunal filter feeders (Collie et al. 2000; Tillin et al. 2006). Heavily trawled areas tend to have fewer filter-feeding, sessile and large-bodied species and more mobile animals, infauna, and scavenging invertebrates (e.g., Tillin et al. 2006; Callaway et al. 2007; Rumohr and Kujawski 2000). Trawling, combined with the erosive power of loosened sediments, destroys the spatial complexity and heterogeneity of benthic communities (Hall 1999; Hily et al. 2008) that derives from the relatively small physical features provided by sponges, empty shells, etc. High habitat heterogeneity supports a variety of peribenthic food that facilitates the survival of juvenile, demersal fish (Kaiser et al. 2002). Structural complexity appears to support the survival of roundfish (Kaiser et al. 2002) whereas the open habitat created by trawling supports flatfish that hide from predators by burrowing in sediments and feeding on infauna (Hall 1999), and even chemosensory flatfish, such as the Dover sole, over visual predators, such as dab and plaice (Kaiser et al. 1999).

A shift in benthos in the North Sea may be attributed to trawling, but the timing of the shift is uncertain: early twentieth century (Rumohr and Kujawski 2000); started before the 1950s (De Vooys and Van der Meer 1998); and prior to 1920 (Frid et al. 2000). Callaway et al. (2007) examined shifts in benthos between 1902–1912 and 1982–1985 and posited that changes might pre-date data. Landings data presented by these authors (their Figs. 2, 3) show different trends for the southern North Sea cf. the central and northern North Sea regions. The relative constancy in flatfish landings for the southern North Sea, over and above climate variability and for a region with a long history of trawling, suggests that changes to benthos occurred around or even before 1900.

**Fig. 2:**
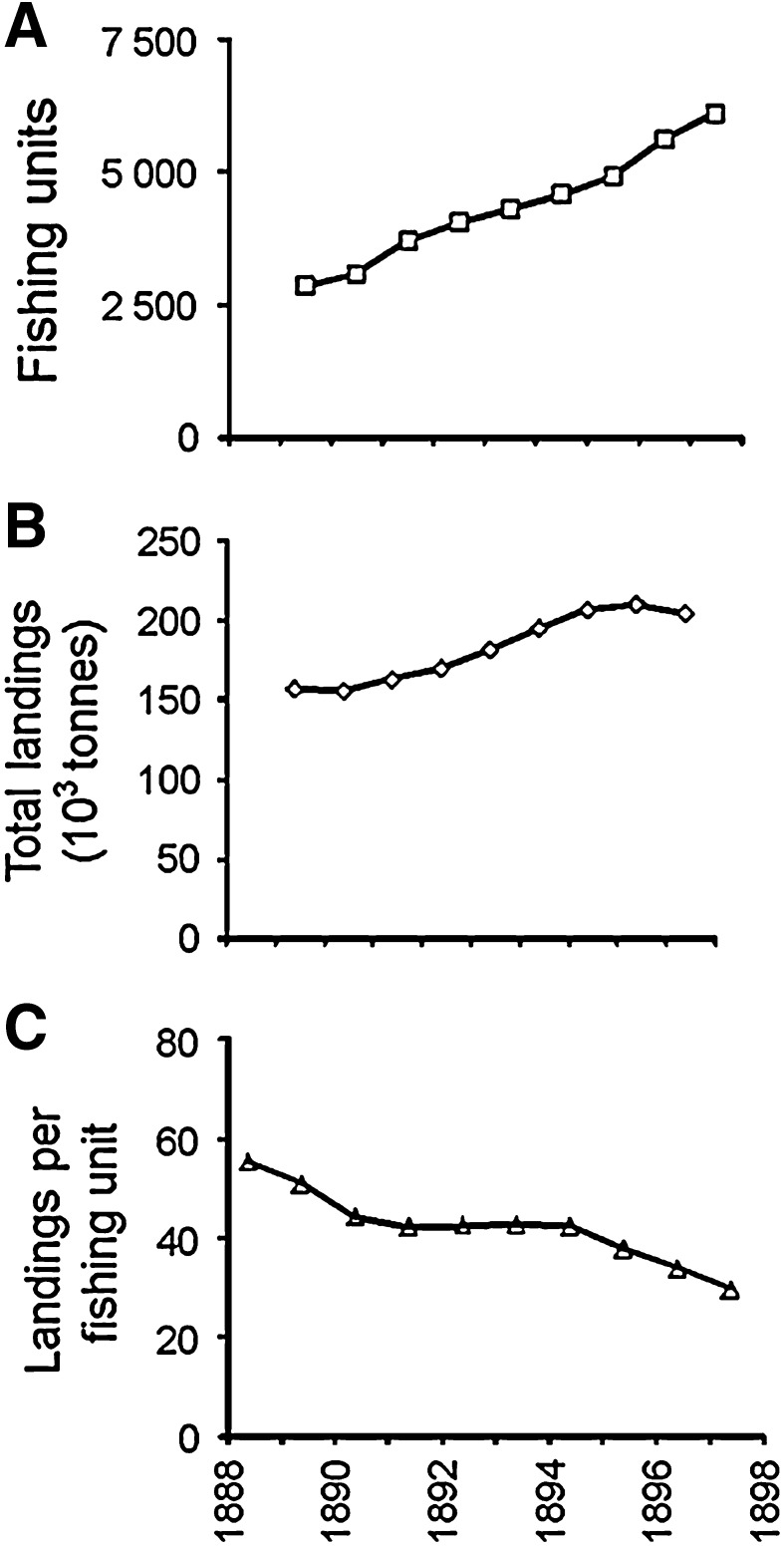
Trends in the fishing fleet, east coast UK (including Grimsby), 1888–1898: **A** catching power (fishing units apply a 4:1 ratio to accommodate the greater efficiency of steam trawlers over smacks), **B** landings of demersal fish (ton), C average catch per fishing unit (Table VIII, Garstang 1903)

**Fig. 3:**
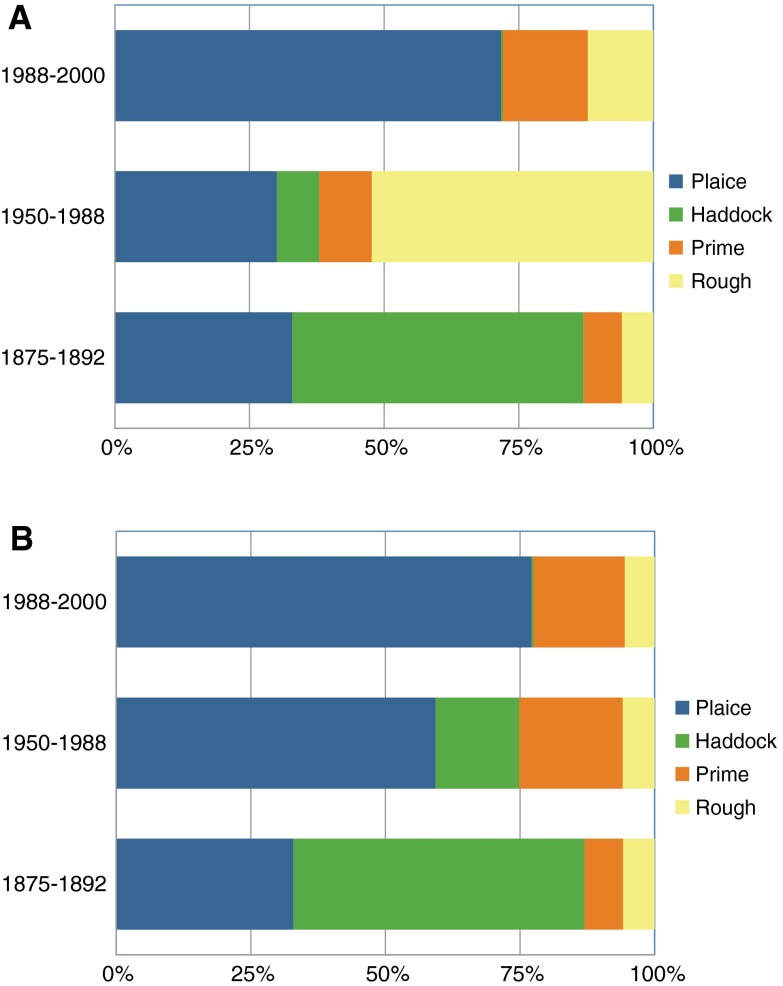
Proportions of different fish classes in landings from the North Sea at Grimsby (Garstang 1903) and at English and Welsh ports (FAOSTAT); “prime” comprises soles, turbot, and brill, and “rough” comprises lemon soles, dabs, and cod (Garstang 1903); **A** cod landings included in rough for 1988–2000 and 1950–1988, **B** cod landings excluded from rough for 1988–2000 and 1950–1988 because cod landings might have comprised a very minor part of rough (based on landings at Lowestoft reported in Garstang (1903))

### Anecdotal Evidence of Trawling Damage in the Nineteenth Century

There is historical and anecdotal evidence for trawling damage, with a possible shift in benthic communities, in the mid- to late-nineteenth century. Fishers were well aware of the damage caused by demersal trawling (Table 2) but attempts to restrain trawling show a long history of failure (Anon 1884; Beaujon 1884; De Groot 1984). UK Royal Commissions between 1866 and 1883 addressed allegations of trawling damage (Huxley 1883). These commissions mark the beginning of the industrialization of trawling. Factors such as competition (“the various classes of fishermen abused the means used for the capture of fish by other classes”—Anon 1884, p. 37), the common property nature of the resource, knowledge constraints, and the widely held view that fisheries were inexhaustible ultimately gave trawling a free rein.

**Table 2:** Selection of quotes from the Trawling Commission 1883 regarding the damage done by beam trawls

	Quote	Source
1	[narrow-meshed nets] proceeding fry of fish are killed, while [the trawl net] destroys the spawn, and even the bait on which fish live”	Beaujon (1884, p. 153)
2	A great deal of the destruction of immature fish by the trawler was caused by the small mesh in the cod end of the net, owing to the ground-chain nipping the ground, and so taking everything into the net	Anon (1884, p. 10)
3	… the reason some grounds were not so prolific as formerly was, first, in consequence of their being overfished; secondly, because of the destruction of the food of the fishes; and thirdly, through the destruction of the fry	Anon (1884, p. 27)
4	… because in the passage of the trawl over the ground, no matter what came in the way it was bound to catch it, and the speed the vessel … caused the meshes to be drawn so tightly that it was impossible for the small fish to escape	Anon (1884, pp. 31–32)
5	… he came to the conclusion that the continual trawling and ploughing up of the ground caused worms and small shell-fish to come to the surface, so as to afford more food to the fish, and that … some good was produced as well as evil	Anon (1884, p. 32)
6	[the trawl] cleans away everything before it	Anon (1885) (see Roberts 2007, p 146)
7	..the result of the process has been that the whole coast of Durham, Northumberland and Yorkshire has been destroyed, till there is nothing left but a mere remnant	Anon (1885) (see Roberts 2007, p. 149)
8	Q. [what] did you see … caught? A. Spawn, coals, boots, shoes, shirts, all kinds of rubbish; little trays [trees] that the fish resorts among; If you saw a little coral, I believe the bottom of the sea is something similar. There is a herbage that the fish live among; it is like a plantation at the bottom…	Anon (1885) (see Roberts 2007, p. 153)
9	…when we used to go for haddocks we used to get all kinds of curiosities, little trays [trees] of all sorts, and every description of shells, and what not. We cannot get anything on the lines now. We used to get things they called coxcombs, and the trawlers have swept them all away the same as they have swept away all the best fishing…^b^	Anon (1885) (see Roberts 2007, p. 153
10	…they have taken away the upper crust of the ground. And, mark you, it is the upper crust that the clams and scallops live amongst …. Q. What was this crust? A. The ground that the scallops live amongst. It is just a ground made up of broken shells, and the like of these sort of things; and underneath that is mud	Anon (1885) (see Roberts 2007, p. 154)
11	The fish have been taken away by the trawlers; the trawlers have destroyed the ground to which these fish came. Q. You think they destroyed it? A. They dragged up the herbage that these fish came to feed upon at certain seasons of the year. The haddock is as fond of dulse [seaweed] and what grows on the bottom as of any food.^c^	Anon (1885) (see Roberts 2007, p. 154)
12	… the ground abounded with small shell fish, particularly the cray fish, which is the chief food of large fish … Now the ground is cleaned of this sort of shell fish by trawling, and now we have no large fish because their food is all taken away	Anon (1885) (see Roberts 2007, p. 155)

^a^This could refer to the spawn of invertebrates or to invertebrates themselves such as sponges and ascidians

^b^This quote as evidence that the bycatch of invertebrates and plants had declined simply because there was less left to gather

^c^While wrong about what haddock eat, this quote points to haddock’s association with complex bottoms and fishers’ knowledge of this

Steam power increased trawler efficiency during the mid-nineteenth century, but major expansion occurred in the last two decades as steam replaced sail (Ansell 1884; Engelhard 2008). The first steam-powered trawlers of the early 1880s were estimated to be 2.6–4.6 times more efficient than sailing trawlers (smacks), but this factor had increased to 5 by 1889, and to 5.5 by 1893 (Garstang 1903). Steam trawlers could deploy otter trawls that could operate over rougher ground, which expanded fishing grounds (Anon 1884; Engelhard 2008).

Figure 2A shows the rapid growth and increasing efficiency of the trawler fleet. Landings (Fig. 2B) show an increasing trend, possibly stabilizing mid-1890s, but landings per fishing unit (Fig. 2C) show a decline (Garstang 1903). Reduced landings per haul were compensated by more hauls, by expanding fishing grounds, and by retaining less desirable fish (Garstang 1903; Roberts 2007). Rumohr and Kujawski (2000) report a decline in landings around the end of the nineteenth century, with haddock landings down by a factor of 5 between 1887 and 1903. However, considerable care must be taken with catch data due to inconsistencies in classification and irregular reporting (Garstang 1903). Routine investigations were begun with the establishment of the International Council for the Exploration of the Sea (ICES) in 1902.

By 1890, much of the greater North Sea had been trawled (Collins 1889); by 1900, some 260 000 km^2^ were being trawled twice per year (Hérubel 1912; Roberts 2007). Despite the limits to available data, and a relatively light trawling frequency (Rijnsdorp et al. 1998 report 8–10 times per year in some parts of the North Sea), it is reasonable to assume that little of the biophysical structure of benthic communities would have remained and that their functioning would have been impaired.

The Oyster Grounds provide a particular instance of the decimation of a benthic community by fishing. The European flat oyster (*Ostrea edulis*) was once very abundant and a staple in diets during the Industrial Revolution. Overfishing saw the decline of inshore beds (Hubrecht 1883). Around 1880, a vast bed of some 25 000 km^2^ was discovered on the Oyster Grounds at a depth of 32–41 m (De Vooys et al. 2004). We estimate that some 400 million oysters were harvested between 1890 and 1910 (based on data in De Vooys et al. 2004). The last living oyster was reportedly taken in the 1970s (OSPAR 2008). The bed was so heavily fished that oyster shells did not figure in Cadee’s (1984) analysis of living and dead macrobenthos.

## Loss of Ecosystem Functioning with Trawling Disturbance

This section builds on “Substantiation of the then Vision” section to assess how southern North Sea benthic communities might have functioned in a relatively undisturbed state, providing some substantiation for the Then vision. We focus on four functions: filtration, sedimentation, secondary production, and trophic complexity.

### Past Functioning of the Oyster Grounds

Oysters are suspension feeders. The most frequently documented function of oyster beds is filtration (e.g., Cloern 1982). We have reconstructed the past filtering capacity of the *Ostrea edulis* bed on Oyster Grounds (Electronic Supplementary Material Table S2) based on accounts of historical densities and current hydrodynamic conditions. All estimates are conservative except perhaps for individual filtration rate, which takes the median of rates reported in the literature. This literature reports on cultured individuals 2–3 years old whereas the larger and older individuals on the Oyster Grounds were possibly more efficient (Rodhouse 1978). We also include a factor to accommodate the filtering capacity of other bivalves, sponges, ascidians, and barnacles reported living between oysters (De Vooys et al. 2004). Our estimates suggest that individual oysters would have cleared some 25 cm of overlying water every day. Bottom waters probably had the highest concentrations of suspended matter (e.g., Van Raaphorst et al. 1998). Given a residence time of the summer-stratified water over the Oyster Grounds of approximately 50 days (Weston et al. 2008), we estimate that this past oyster bed was able to filter the bottom 15 m of the water column completely, in the process retaining fresh organic matter advecting from the southern North Sea.

Filtration is a top-down grazing control on phytoplankton in which energy flows are directed from pelagic to benthic food chains (French McCay et al. 2003). Loss of suspension feeders may contribute to increased phytoplankton stocks and turbidity (e.g., Jackson et al. 2001). Conversely, increased water clarity and a shift toward a more benthic-dominated system has occurred with the establishment of suspension feeders, such as the zebra mussel, *Dreissena polymorpha* (Stewart and Haynes 1994; Fahnenstiel et al. 1995), the Asian clam, *Potamocorbula amurensis* (Nichols et al. 1990), and the blue mussel, *Mytilus edulis* (Russell et al. 1983). By incorporating carbon and nutrients from suspended particles in their body mass for further trophic transfer and/or in situ benthic processing, oysters and their associates regulate nutrient reflux and pelagic productivity.

*Ostrea edulis* is a biogenic reef-forming species. Biogenic reefs stabilize and accumulate sediments, provide hard substrata and complex habitat for colonization by flora and fauna (Holt et al. 1998). The sandy sediments prevalent in the southern North Sea (Kröncke et al. 2004; Schlüter and Jerosch 2008) are subject to high or frequent hydrodynamic disturbances. Ecosystem engineers, such as the tube worm *Lanice conchilega* (Borsje et al. 2009), enhance bed stability as well as provide shelter for juvenile flatfish. The long-lived, subtidal oyster beds should have functioned in a similar fashion. The diversity of species and of feeding types reported by De Vooys et al. (2004) suggests trophic complexity. The benthos of the Oyster Grounds and the southern shallower transition area of the Frisian Front is currently dominated by the burrowing brittlestar *Amphiura*, the deposit feeding ghost shrimp *Callianassa* and the mollusc *Abra* (Van Raaphorst et al. 1998; Dauwe et al. 1998; Kröncke et al. 2004). We conclude that loss of the oyster and its associates has reduced benthic diversity and benthic functioning.

### Extrapolation Beyond the Oyster Grounds

Oysters were once abundant in estuaries, near coasts and presumably located to take advantage of riverine sources of particulate organic matter. Extrapolation of benthic functioning from oyster beds to southern North Sea benthic communities as a whole is constrained by our limited knowledge of species composition, structure, and extent. Quotes 7–12 in Table 2 support the notion of a “living crust” on the bottom of the southern North Sea (Roberts 2007). Evidence for a widespread decline in filtration function can be derived from the biotic traits of species whose distribution in the greater North Sea changed over the last 100 years due to trawling (Callaway et al. 2007). We observe that organisms feeding on particulate organic matter suspended in the water column have declined whereas scavengers/predators have increased (Electronic Supplementary Material Table S3).

## From Benthic Functioning to Ecosystem Services

Ecosystem functioning underpins ecosystem services, defined as “aspects of ecosystems utilized (actively or passively) to produce human well-being” (Fisher and Turner 2008). We identify five ecosystem services from the southern North Sea that are supported by benthic functioning. The first is “resistance and resilience,” the ability of the ecosystem to resist and subsequently to recover from disturbance and so to continue delivering ecosystem services. Strictly speaking, this service is a supporting service (MEA 2003), with its significance estimated in its contribution to other, final services. The second service is “transparent water,” benefitting recreation. A Secchi depth exceeding 1 m is required for the Blue Flag certification of beaches; Blue Flag standards are incorporated in the EU Water Framework Directive (Directive 2000/60/EC). The third service is the “processing of nutrients.” The southern North Sea receives nutrient surpluses and wastes from a variety of human activities such as agriculture and wastewater treatment. These activities avoid the costs of other means of disposal. The fourth service is “food production,” with direct benefits to fisheries. The fifth service is carbon storage and sequestration. Biogenic reefs represent a carbon sink and their growth sequesters carbon, which counters global warming to the benefit of humans. This section draws on how benthic functioning in the two visions affects their provision of ecosystem services.

### Resistance and Resilience

The MSFD requires that ecosystems are returned to a healthy state that maintains their resilience to human-induced environmental change (MSFD Article 3(5)). The Then vision, in re-creating past functioning, represents a presumably healthy marine ecosystem that would display resistance and resilience to disturbance. The Now vision does not correspond to a healthy ecosystem but its persistence suggests a degree of resistance, even resilience. It could be a new stable state, or hysteresis may be delaying recovery. Resistance and resilience are closely linked to vigor and organization when assessing ecosystem health (Mageau et al. 1998). These two concepts are used to distinguish the two visions and to argue that the Then vision provides better guarantees for the continued supply of ecosystem services.

Vigor relates to energy flow through an ecosystem (Odum 1971) and to its scope for growth (Costanza 1992). An ecosystem needs vigor to recover from disturbance by recolonisation and population growth. Primary production is a component of vigor. In a healthy ecosystem, it is coupled to consumption (Tett et al. 2007). Primary production is decoupled from consumption in the southern North Sea as evidenced by an increase in algal blooms, including harmful blooms (Cadée and Hegeman 2002; OSPAR 2010). Decoupling has been achieved by removing consumers (suspension feeders) combined with rapid nutrient recycling augmented by anthropogenic eutrophication so that production outpaces pelagic consumption. The excess of nourishment in the Now vision means that its vigor is unlikely to support recolonisation and population growth; this vision is likely to be less resilient.

The organization of an ecosystem comprises its biodiversity, its food web, and its biophysical structure. Marine shallow-water benthos is thought to function well only when all expected guilds are present (Bolam et al. 2002), a situation that can be expected in the Then vision. Ecological theory holds that ecosystems with damaged organization have little resilience. The Now vision has lost at least two benthic guilds—ecosystem engineers and suspension feeders. A shift to a simpler food web based on pelagic production seems likely (as is argued in “Past Functioning of the Oyster Grounds” section, but see also Christensen and Richardson 2008). A simpler food web would make the Now vision more vulnerable to secondary extinction and fragmentation, and so less resistant to disturbance (Dunne et al. 2002; Gilbert 2009). Further, the diversity resistance hypothesis argues that diverse communities are highly competitive and readily resist invasion (Levine and D’Antonio 1999), although empirical evidence is mixed (Stohlgren et al. 2003). The Then vision could be more resistant to invasion.

### Water Transparency

Filtration and sedimentation by benthic communities dominated by suspension feeders caters for a relatively transparent water column in the Then vision. The absence of these functions and the prevalence of trawling mean that The Now vision is characterized by turbidity. The main beneficiary of water transparency is recreation in the coastal zone. Coastal waters are naturally turbid due wave and current action and sediment loads in river plumes. Poor transparency has not caused beach closures in recent decades, so any improvement is unlikely to generate additional benefits. This ecosystem service does not differ in the two visions.

### Processing Anthropogenic Nutrient Surpluses

Direct anthropogenic sources of nutrients to the southern North Sea comprise ~25% of total influx (Vermaat et al. 2008). Pelagic primary production in the southern North Sea is currently light-limited (McQuatters-Gollop et al. 2007), with chlorophyll showing increasing trends despite declining nutrient availability (McQuatters-Gollop et al. 2009). The Now vision is typified by an abundance of nutrients in surface waters. Nutrients are diluted, exported, or contribute to algal growth that may be uncoupled from secondary production. Recycling of nutrients is relatively rapid. In the Then vision, nutrients fixed by phytoplankton are retained in benthic communities, which ensure coupling of primary and secondary production as well as modulating nutrient reflux and keeping pelagic primary production nutrient-limited. In contrast to the Now vision, the Then vision actively processes nutrients.

The benefits from this service are unclear. Activities releasing nutrients benefit whether or not nutrients are processed. Processing benefits coastal activities, such as tourism and aquaculture (OSPAR 2010), that currently suffer from algal blooms. Bloom damage is typically a coastal phenomenon, responding to river nutrient loads (Lancelot et al. 2007). Restoration of benthic communities in estuaries and inshore zones is needed to intercept this source. Nutrients in Dutch and Belgian coastal waters also derive from UK sources. Los (pers. comm.) estimated this contribution in 2002 to be ~5%. Only small benefit can be expected in the Then vision from intercepting UK nutrients.

### Production of Food

Both visions produce food from catching demersal fish. We distinguish the two visions in the diversity of demersal species. Data from Garstang (1903) on trawler landings at Grimsby are used as a proxy for the Then vision. UK landings 1950–1988 and 1988–2000 from ICES box IVc (FAO 2012) are used to illustrate the Now vision. Differences in the compositions of landings are evident in Fig. 3. Figure 3A shows that the proportion of haddock has declined, while that of rough increased, particularly between 1875–1892 and 1950–1988. Cod dominates this category (>90%) in the FAO data series, and the increase in rough probably reflects the gadoid outburst in the late 1960s–1970s (Daan 1978).

Cod landings at Grimsby were not specified, but landings at Lowestoft (cod <0.1% of total landings, Garstang 1903) suggest that cod may have been a minor component of catch. Figure 3B excludes cod from landings 1950–1988 and 1989–2000. The proportion of haddock still declines, but so does rough; proportions of plaice and possibly of prime fish increase. The common sole (*Solea solea*) comprises ~80% of prime fish in the FAO data series, and so a growing share of sole is possible. Rijnsdorp and Vingerhoed (2001) suggested that beam trawling might have improved the feeding conditions for these species by enhancing the abundance of small opportunistic benthic species, such as infaunal polychaetes.

Production of food in the Now vision focuses on flatfish that have benefited from changes to benthic communities caused by trawling; plaice dominate demersal landings by weight, but sole is the more valuable fish (O’Higgins and Gilbert 2014). In the Then vision, demersal stocks are more diverse and comprise a variety of flat- and roundfish such as haddock, brill, turbot, lemon sole, dab, possibly halibut, as well as plaice, sole, and cod.

### Carbon Sink and Sequestration

Some of the carbon ingested by species forming biogenic reefs is fixed in their structures. Since oysters grew on oysters, the bed on the Oyster Grounds would have represented a sink of carbon that we estimate was of the order of 7–8 million ton C. This stock and associated capacity for carbon sequestration have been lost. This service is not provided in the Now vision. In the Then vision, bivalve populations and biogenic reefs are abundant. However, both the formation of CaCO_3_ structures and their contribution to carbon sequestration in the future are in doubt as ocean acidification threatens the ability to maintain such structures (Gazeau et al. 2007 and others). Further, the net effect of sequestration is not straightforward as precipitation of CaCO_3_ produces CO_2_ which might reduce seawater’s capacity to take up CO_2_ from the atmosphere. Pelejero et al. (2010) estimates that, in today’s oceans, current conditions favor net carbon sequestration. The Then vision has only a limited window of opportunity to sequester carbon.

## The Societal Choice

Our analysis shows that the benefits from the Then vision derive primarily from restoration of ecosystem resistance and resilience, and their support for continued supply of ecosystem services. Some benefit would derive from a greater variety of demersal fish and mitigation of damage from harmful algal blooms. These benefits would be offset by costs. Given that trawling caused loss of benthic functioning, its restoration means restraints on trawling to facilitate restoration of benthic functioning and to prevent repeating history. Alternative fishing techniques, perhaps with Marine Stewardship Council certification, and/or careful delineation of trawling grounds to, for example, maintain habitat supporting valuable sole stocks might be required. There are considerable uncertainties as to how quickly the Then vision might be realized, if it can be realized at all (e.g., Duarte et al. 2009), and so the cost to fisheries is difficult to estimate. The Then vision involves a large marine ecosystem experiment that could take decades with few, if any, intermediate criteria for assessing progress.

Despite inherent uncertainties with the Then vision, we contend that it is germane to current discussions on GES. The Then vision provides perspective on what a relatively undisturbed state of the southern North Sea might be. As a long-term goal, it would give focus for specification of GES, whether or not targets are achieved by 2020. Sea-wide degradation of the southern North Sea was triggered by industrial trawling. While now subject to a wider diversity of anthropogenic pressures, any discussion of GES, any measure to achieve it, is moot if this source of degradation is not tackled.

It is not our purpose to advocate the Then vision. GES is, ultimately, a societal choice. Society may choose for a status that is little different from the Now vision because the uncertainties of achieving better engender costs that are not outweighed by benefits. Our analysis shows that such a choice maintains the southern North Sea as a turbid fishpond producing flatfish, would indicate a willingness-to-accept damage from algal blooms, and would carry the risk of further state changes that could threaten supply of ecosystem services as a result of poor resistance and resilience.

The choice is not simply between Now and Then as there are potentially any number of intermediates, although it is entirely possible that Now and Then broadly describe two relatively stable states so that small interventions (e.g., further decreases in anthropogenic nutrient loading) would have only marginal benefits. We have also ignored the effects of climate change. Climate change poses a threat to the Now vision as cod and plaice are Boreal species. Based on studies of their recent response to warmer conditions (Rijnsdorp et al. 2009; Heath et al. 2012), their abundance and distribution in the southern North Sea are likely to be curtailed as the sea warms. Sole is a Lusitanian species and, while expected to increase, it has exhibited significant negative relationships between temperature and recruitment, much like cod and plaice (Cook and Heath 2005). A choice for the Now vision also carries uncertainties about future delivery of food production services. The Then vision could be seen to widen our choices given the uncertain future of fish stocks.

The effects of past and present pressures on ecosystems generate considerable uncertainty about setting and reaching targets for GES. Our contrasting of two visions is intended to stimulate debate about the kind of North Sea society wants. Whatever vision emerges will need to be robust in the light of foreseen and unforeseen pressures from an increasingly globalized North Sea.

## Electronic supplementary material

Below is the link to the electronic supplementary material.

Supplementary material 1 (PDF 387 kb)
