# Supplementary material for: Visions for the North Sea: The Societal Dilemma Behind Specifying Good Environmental Status
Source: Ambio. 2014 May 24;44(2):142–53. doi: 10.1007/s13280-014-0536-5 (PMC4329130; doi:10.1007/s13280-014-0536-5)
Supplement: Supplementary file 1 — Supplementary material 1 (PDF 387 kb) [file 13280_2014_536_MOESM1_ESM.pdf]

AMBIO
Electronic Supplementary Material

Visions for the North Sea: the societal dilemma behind specifying Good Environmental Status

Alison J. Gilbert, Abigail McQuatters-Gollop, Olivia Langmead, Laurence Mee, Jan Vermaat

Electronic Supplementary Material:

Table S1. Elaboration of ecosystem functions and their relevance for benthic ecosystems

**Function**	**Definition**	**Relevance for benthic ecosystems**
Sedimentation	The act or process by which sediments deposit	Deposition of particulate organic carbon, stabilization of sediments and prevention of erosion, fixes nutrients in sediments for storage and/or further processing
Filtration	The process of separating particles from the fluid in which they are suspended	Effect of filter and suspension feeders
Primary production	The generation of organic compounds, usually via photosynthesis	Autotrophs fix carbon for further trophic transfer, photosynthesis is a source of oxygen
Secondary production	The rate of generation of biomass by heterotrophs	Carbon and nutrient transfer through benthic food webs, interception of carbon settling from pelagic zones
Trophic complexity	The complexity of ecosystem feeding relations	Measured by mean trophic index or mean trophic level (TL) (Pauly et al. 1998; Pauly and Watson 2005)
Nutrient exchange	The movement of nutrients among biota, between biota and the physical environment, and its import to and export from ecosystems	Role of benthic organisms in transferring nutrients through food webs, influence on microbial production, exchange among water bodies
Recruitment	The increase in a natural population as progeny grow and new members arrive	Of special relevance for demersal species of commercial importance

Table S2. Reconstruction of filtering capacity of the oyster community on the Oyster Grounds around 1880

Variable	Estimate	Justification	Sources
Density of oysters	5 m^{-2}	Conservative: from clump density (3 m^{-2}) and number of individuals per clump (~3)	De Vooys et al. (2004); OSPAR (2008)
Individual filtering capacity	2 l h^{-1}	Median of 1-3 l h^{-1} for 2-3 year-old individuals, depending on quantity and quality of suspended matter, condition of animal and water temperature; older individuals were probably more efficient and would have been more prevalent in the Oyster Grounds; clogging is not likely given that current suspended matter concentrations are generally <2 mg l^{-1} during the growing season	Rodhouse 1978; Van Raaphorst et al, (1998); Riisgård and Larsen (2000); Barnabé & Barnabé-Quet (2000); Cugier et al. (2010)
Volume filtered by oysters per unit area	0.24 m^3 m^{-2} d^{-1}	0.002 (m3 h-1) x 5 (m2) x 24 (h)	
Volume filtered by oysters and associated community per unit area	0.5 m^3 m^{-2} d^{-1}	Factor 2 as an estimate. Other filter feeders are known to have been abundantly present; while individual associates had a lower filtration rate, together they may have formed a substantial filtration capacity	De Vooys et al. (2004); Riisgård and Larsen (2000)
Depth of the water layer that should be processed	15 m	Oyster grounds are stratified during summer; bottom waters are richer in organic particulates and algae between 25 and 40 m	Figure 4 in Van Raaphorst et al (1998)
Time needed to filter this water column	30 d	15 (m) / 0.5 (m^3 m^{-2} d^{-1})	
Residence time of water over Oyster Grounds during summer	50 d	Particularly spring tidal currents will resuspend upper sediments but resettlement is rapid. Mean modeled flushing time is 33 days, range 12-53 days	Lenhart et al (1995), Van Raaphorst et al, (1998)

Table S3. Feeding method (SF = suspension feeder, DF = deposit feeder, S/P – scavenger/ predator), food and growth form of benthic invertebrate species whose distribution in the North Sea changed[a] between 1902-1912 and 2000 (benthic invertebrate species derived from Table 2, Callaway, 2007; biotic traits derived from MarLIN 2006)

	Species Name	Feeding method	Food type	Growth form
≥50% reduction in spatial presence	*Aequipecten opercularis*	SF	suspended particulate matter, plankton	bivalve
	Anomia ephippium	SF		
	Arctica islandica	SF, DF	phytoplankton	bivalve
	Brissopsis lyrifera	DF	detritus, foraminifers, small organisms in sediment	globose
	Echinocyamus pusillus	DF	detritus	globose
	Modiolus modiolus	SF	bacteria, phytoplankton, detritus, dissolved organic matter	bivalve
	Ophiothrix fragilis	SF, DF	phytoplankton	radial, stellate
	Phaxas pellucidus	SF		bivalve
	Pisidia longicornis	SF		articulate
	Spatangus purpureus	DF	detritus	radial, globose
	Velutina velutina			
≥100% increase in spatial presence	*Corystes cassivelaunus*	P/S	burrowing invertebrates	articulate
	Ebalia spp.			
	Echinocardium cordatum	DF	detritus	globose
	Liocarcinus depurator	P/S	polychaetes, crustaceans, molluscs, ophiuroids, fish	articulate
	Liocarcinus holsatus	P/S	carrion, invertebrates (e.g. polychaetes, small bivalves, echinoderms, small crustaceans), juvenile fish	articulate
	Ophiura ophiura	P/S	small bivalves, polychaetes, crustaceans, carrion	radial, stellate

a The change in distributions of another 18 species was minor or unclear. A chi-square contingency test (two-tailed) was used to compare the feeding types in the above groups and showed a significant difference: χ^2=12.88, degrees of freedom=2, *p*=0.0016.

References for ESM

1. Barnabé, G. and R. Barnabé-Quet. 2000. Ecology and management of coastal waters: the aquatic environment. Chichester, UK: Praxis Publishing Limited.
2. Cugier, P., C. Struski, M. Blanchard, J. Mazurié, S. Pouvreau, F. Olivier, J. R. Trigui, and E. Thiébaut. 2010. Assessing the role of benthic filter feeders on phytoplankton production in a shellfish farming site: Mont Saint Michel Bay, France. Journal of Marine Systems 82: 21-34.
3. Lenhart, H.J., G. Radach, J.O. Backhaus and T. Pohlmann. 1995. Simulations of the North Sea circulation, its variability, and its implementation as hydrodynamical forcing in ERSEM. Netherlands Journal of Sea Research 33: 271-299.
4. MarLIN. 2006. BIOTIC - Biological Traits Information Catalogue. Marine Life Information Network Plymouth: Marine Biological Association of the United Kingdom. Retrieve 8 October, 2012, from http://www.marlin.ac.uk/biotic
5. Pauly, D., Christensen, V., Dalsgaard, J., Froese, R. & Torres, F. Jr. 1998. Fishing down marine food webs. Science 279: 860–863.
6. Pauly, D., and R. Watson. 2005. Background and interpretation of the 'marine trophic index' as a measure of biodiversity. Philosophical Transactions of the Royal Society B-Biological Sciences 360: 415–423.
7. Riisgård, H. U. and P. S. Larsen. 2000. Comparative ecophysiology of active zoobenthic filter feeding, essence of current knowledge. Journal of Sea Research 44: 169-193.
